# Information and communication technology for increasing healthy ageing in people with non-communicable diseases: identifying challenges and further areas for development

**DOI:** 10.1007/s40520-019-01258-8

**Published:** 2019-07-17

**Authors:** Alessandro Monaco, Stefania Maggi, Paula De Cola, Tarek A. Hassan, Katie Palmer, Shaantanu Donde

**Affiliations:** 1 HEC, 1, Rue de la Liberation Jouy en Josas, Paris, France; 2 CNR-Neuroscience Institute, Aging Branch, Padua, Italy; 3 Pfizer Inc, New York, NY USA; 4 Oliba, Rome, Italy; 5 Pfizer Ltd, Surrey, UK

**Keywords:** Chronic disease, Ageing, NCD, Clinical practice, Successful ageing

## Abstract

Information and communication technology (ICT) within healthcare covers a range of technologies that aim to improve disease management or help modify health behaviors. We discuss clinical practice and system-related ICT challenges in Europe in relation to healthy ageing in people with non-communicable diseases (NCD). Although ICT use within healthcare is increasing, several challenges remain, including: (i) variations in ICT use within Europe; (ii) under-use of electronic health records; (iii) frequent use of single domain outcomes; (iv) shortage of clinical trials on current technologies; (v) lack of involvement of patients in ICT development; (vii) need to develop and adapt ICTs for people with cognitive or sensory impairment; and (viii) need to use longitudinal big data better. Close collaboration between key stakeholders (academia, biopharmaceutical and technology industries, healthcare, policy makers, patients, and caregivers) should foster both technological innovation and innovative models to facilitate more cost-effective approaches, ultimately leading to increased healthy ageing.

## Active and healthy ageing

Currently, almost a fifth of the European population is made up of people over the age of 65, with an increase of more than 2% in the last decade [1]. The number of Europeans aged over 65 is forecast to double in the next 50 years. Thus, ageing is a priority area for European policy makers and healthcare providers. As life expectancy increases an important question arises—how many people live an active and healthy life in older age? The World Health Organization (WHO) defines healthy ageing as “the process of developing and maintaining the functional ability that enables well-being in older age” [2]. The epidemiological transition that has occurred is reflected in a decrease in communicable diseases and an increase in the prevalence of non-communicable diseases (NCD) and mortality from NCDs [3, 4]. This is further exacerbated by lifestyle factors that increase the risk of multiple age-related NCDs [5]. In the 2030 Agenda for Sustainable Development, WHO described a goal to reduce premature mortality from NCDs by one-third by 2030 through prevention and treatment, as well as to promote mental health and well-being [6]. Addressing barriers to healthy ageing is becoming an important issue in Europe. Solutions may include prevention, appropriate management of NCDs, and slowing of functional decline. Importantly, a life-course approach is needed that recognizes the influence of early- and mid-life factors and addresses NCD prevention and management at the earliest stages possible [7, 8].

## Project CHANGE

Project CHANGE (Clinical practice-oriented cHange solutions towards Active aNd healthy aGEing) is a new initiative that aims to identify solutions to increase healthy ageing and reduce the burden of NCDs. It involves an institutional collaboration between Pfizer and the European Innovation Partnership on Active and Healthy Ageing (EIP on AHA). EIP on AHA was launched by the European Commission to foster innovation and digital transformation in the field of active and healthy ageing and strengthen EU research and innovation by bringing together a range of relevant actors in the field. One objective of Project CHANGE is to identify clinical practice- and system-related challenges and gaps in Europe in relation to healthy ageing in people with NCDs.

## Priority areas for older people with NCDs

Project CHANGE has identified several priority areas for fostering healthy ageing in older people with NCDs. These include the development and increased use of integrated care models; enhancing shared decision-making; increasing awareness and education on ways to prevent and manage NCDs at the patient, family, and caregiver level; increasing patient engagement and empowerment; promoting comprehensive assessment and follow-up of mobility as well as pain; improving treatment adherence interventions; and enhancing education and training in geriatric medicine within the existing core-curriculum for health practitioners. One enabling area identified as a major priority was the use of information and communication technology (ICT) for assessment, management, and integration as well as follow-up of older people with NCDs.

## ICTs for healthy ageing: current use and challenges

The use of ICTs in healthcare systems is rapidly increasing. This is partly due to a growth in the development and availability of ICT healthcare tools, but also because ICT usage is increasing in older individuals [9]. ICTs can provide support for monitoring disease symptoms and searching for medical information, potentially helping the older population to remain active and independent for as long as possible. ICT within healthcare covers a wide range of different technologies that aims to improve disease management or helps to modify health behaviors [10]. Project CHANGE has identified several challenges related to the use of ICTs in the area of healthy ageing (Table 1), which we discuss below.

**Table 1:** Gaps, barriers, and actions needed for a better implementation of information and communication technologies (ICT) in older persons with non-communicable diseases (NCD)

Gaps and barriers	Actions needed for a better implementation of ICTs in older persons with NCDs
Variations in ICT use within Europe	Development and use of linked pharmaceutical, medical and care registries at both national and European levels Better use of integrated care models that use ICT to support information sharing between healthcare providers
Under-use of electronic health records	Increased use of electronic health methods in all European countries, ideally with standardized methods
Frequent use of single-domain outcomes	Development of ICTs that combine multiple dimensions (e.g., physical functioning, mental health, and well-being) More extensive use of comprehensive multi-modal IT-based interventions
Shortage of clinical trials on current technologies	Use of different trial types, including randomized control trials and adaptive trials More trials designed to assess multiple outcomes (e.g., clinical outcomes and symptomology, quality of life, functioning, and mental health)
Lack of involvement of patients and caregivers in ICT development	ICT developers need to include the intended users during the creation process (including consultation with healthcare providers, patients, and caregivers)
Lack of ICTs for people with cognitive or sensory impairment	Better customization and design of ICTs for persons with visual or hearing impairment, or persons with cognitive dysfunction Testing of ICTs in diverse patient groups is needed
Lack of longitudinal big data, and issues with fragmented data, privacy issues and data standardization	Better linking of currently available big data at national and European levels Initiatives to decrease data fragmentation Development of policies regarding security and privacy issues

### Variations in ICT use between European countries

The first challenge is that ICT usage varies within European healthcare systems. Some Nordic countries routinely use telemedicine and have, for example, nationwide computerized prescription registries that link data from all the different healthcare providers as well as pharmacies. Some other European countries have under-developed and unlinked registries that make it difficult to provide integrated care for people with NCDs. This is especially relevant for the ageing population, where multimorbidity is common [11] and integrated care models that use ICT to support information sharing between healthcare providers for the management of such patients is recommended [12].

### Under-developed use of electronic health records

A second, related issue is that electronic health records generally need to be better developed and utilized to prevent unneeded concomitant prescriptions and unnecessary prescriptions. This would reduce the burden of inappropriate pharmacy and polypharmacy, which are associated with negative outcomes such as frailty [13]. In patients with chronic pain, it has been demonstrated that combining electronic health records with patient-reported outcomes can help determine which treatments are most effective for these patients [14]. Electronic health records are also invaluable tools for surveillance and can help to increase patient empowerment by providing individuals with access to their own clinical information.

### Single-domain outcomes

There has been a surge in the development of ICTs for measuring symptoms and treatment adherence, as well as management of adverse drug reactions and polypharmacy. Many ICTs focus on these individually as single measures and outcomes. For example, self-care Apps for monitoring hypertension often evaluate single signs/symptoms such as blood pressure or lifestyle behaviors such as weight loss or smoking cessation. However, multiple outcomes might be clinically relevant to people with NCDs, especially in the presence of multimorbidity [15]. Similarly, because hypertension can be a symptom of another underlying pathology, such as atherosclerotic renal artery stenosis, renal failure, metabolic syndrome, or hypothyroidism [16], an affected person may need to track multiple signs and symptoms. For example, it could be useful to use ICT to simultaneously track blood glucose, weight, triglyceride levels, and high-density lipoprotein in people who suffer from hypertension related to a metabolic syndrome. Further, healthy ageing encompasses multiple dimensions including physical functioning, mental health, and well-being [2]. Thus, disease-specific ICTs that focus on a single or limited number of factors may have minimal value to people with more than one chronic condition or medicine. These individuals may need to utilize a number of different Apps to monitor their health. Therefore, comprehensive and coordinated multi-modal IT-based interventions have the potential to support positive health changes in older individuals.

### Lack of clinical trials

Current ICTs in the health arena are not routinely regulated and few have undergone clinical testing in well-designed trials. The development of ICTs is growing rapidly but their efficiency and cost-effectiveness need to be tested in large, randomized-controlled clinical trials in relevant groups of people. Other designs such as adaptive trials may also provide useful information. Different outcomes that are germane to aspects of healthy ageing and relevant to ICT need to be assessed. These could include symptoms and disease progression, adherence to treatment plans including medication when relevant, as well as adherence to follow-up visits. The concept of healthy ageing also includes well-being. Consequently, it is essential to move away from simple symptom-based approaches to also include evaluation of how ICTs can help to improve quality of life, functioning, and mental health, even if their primary application focuses on a clinical aspect. It is imperative, that the clinical outcomes along with the quality of life measures are relevant to older people.

### Lack of involvement of patients and caregivers in ICT development

One major challenge is that many of the current ICTs on the market have not been developed with healthcare providers or patients. It is essential that ICTs take into account the needs, abilities, resources, and time of patients as well as healthcare providers. Indeed, access to ICT among the older population may be varied depending on various factors, such as access to technology, education, socioeconomic status, and even their ability and willingness to use technology. ICTs are not always well-integrated into the natural flow of daily activities in clinical or home settings, which is important for achieving long-term sustainability of such technologies. Therefore, it is imperative to have the intended users, older people in this case, as part of the creation process. Co-creation may also facilitate adoption by including older people along with healthcare providers and caregivers in the design of the requisite education and training needed for ICT use.

### ICT use in people with cognitive impairment

Older adults can sometimes be resistant to the use of new technologies, especially if they require learning new skills [17]. Further, people with cognitive impairment, dementia, or sensory deficits may face difficulties in using technology unaided [18], yet these individuals account for a significant proportion of the older population [19]. Thus, ICTs need to be designed or customized to be directly utilized by these groups of people. Indeed, it is equally important to consider how technology can be used not only by people with NCDs but also by their caregivers. For example, ICTs have been shown to aid caregivers who are supporting people with dementia in areas such as increasing effective care coordination, reducing caregiver burden, and decreasing poor mental health outcomes in caregivers [20].

### Use of longitudinal big data

Finally, ICTs need to consider the life-course approach for understanding the risk and development of NCDs. The life-course approach has established that factors occurring at many stages of the lifespan increase the risk of developing NCDs in older ages, from factors occurring in utero to birth and early childhood as well as in adolescence, middle age, and late-life. The Scottish Mental Health Survey 1947 is a worthy example, where longitudinal data has shown the role of childhood IQ on lowering the risk of mortality from certain NCDs in older age [21]. This may be a relevant avenue for ICT to focus on, including the use of longitudinal big data to evaluate individual and combined risks occurring over the lifespan. Such information can be used to design solutions that can be tailored to an individual’s life-course risk profile. There are many opportunities for this, as longitudinal big data is already available [22–24]. Initial applications of big data analytics have proved useful in NCD management, especially in terms of risk prediction, diagnostic accuracy cost, hospital readmission reduction, and patient-outcome improvement, [23]. However, many are not in public archives and, therefore, increased data availability and data sharing are needed, as well as attempts to overcome current obstacles such as fragmented data, privacy issues, and data standardization [24].

## Future research needs

As there are multiple challenges regarding the use of ICT to promote healthy ageing, it is essential to have coherent strategies for the assessment, development, regulation, and use of ICTs in older people. There are several areas that need to be prioritized for future research. First, good quality research should focus on assessing the long-term effects of ICT for disease monitoring, healthcare management, health education, and behavior change. Multiple factors that are relevant to healthy ageing should be evaluated. This could include symptoms of the disease, progression, and adherence to treatment plans as well as factors such as level of functioning, frailty, well-being, and quality of life. Further, large, randomized-controlled trials are needed, especially ones that include the target audiences, in this case older people with multimorbidity or frailty. These individuals are often excluded from clinical drug trials [25] despite the fact that multimorbidity has a high prevalence in older ages [11]. Adaptive trials may also be pertinent to evaluating and enhancing ICT solutions for older people with NCDs. Further, machine learning to predict which people with NCDs are at significant risk of functional decline, frailty, and mortality should be better used. Although numerous instruments exist, they are not extensively employed in clinical practice. In addition, clinical guidelines need to be constantly updated to reflect the rapid advances in technology. Indeed, the recently published European guidelines for the management of hypertension [26], for example, covers a number of new ICT solutions, although evidence-based knowledge on their use is still lacking. Finally, projects that bring together different actors in the field of healthy ageing, from academia, healthcare systems, policy makers, patients and their caregivers, technology developers, and the biopharmaceutical industry are an essential part of moving forward. There are several ongoing projects that are helping to achieve these goals, including the “Active Assisted Living Programme” (AAL), which funds projects that work towards creating market-ready products and services for older people with the goal to increase ageing well in the digital world. Project CHANGE is a collaboration of industry, academic experts, healthcare professionals and patient-representatives aiming to drive initiatives that will address unmet needs in the ageing population. The potential projects supported by the partnerships will also aim to improve ICT implementation in European populations with NCDs. The magnitude and ubiquitous nature of the rise of NCDs, coupled with the demographic shift toward ageing populations, necessitates collective action. Initiatives such as Project CHANGE can help to combine multidisciplinary knowledge from various stakeholders who are interested in achieving the same goal; to increase healthy ageing and reduce the burden of NCDs.
